# Health and Social Needs in Three Migrant Worker Communities around La Romana, Dominican Republic, and the Role of Volunteers: A Thematic Analysis and Evaluation

**DOI:** 10.1155/2016/4354063

**Published:** 2016-08-04

**Authors:** Aaron S. Miller, Henry C. Lin, Chang-Berm Kang, Lawrence C. Loh

**Affiliations:** ^1^University of Toronto, Toronto, ON, Canada M5S 1A1; ^2^The 53rd Week, Brooklyn, NY 11201, USA; ^3^Perelman School of Medicine, University of Pennsylvania, Philadelphia, PA 19104, USA

## Abstract

*Objective.* For decades, Haitian migrant workers living in bateyes around La Romana, Dominican Republic, have been the focus of short-term volunteer medical groups from North America. To assist these efforts, this study aimed to characterize various health and social needs that could be addressed by volunteer groups.* Design.* Needs were assessed using semistructured interviews of community and professional informants, using a questionnaire based on a social determinants of health framework, and responses were qualitatively analysed for common themes.* Results.* Key themes in community responses included significant access limitations to basic necessities and healthcare, including limited access to regular electricity and potable water, lack of health insurance, high out-of-pocket costs, and discrimination. Healthcare providers identified the expansion of a community health promoter program and mobile medical teams as potential solutions. English and French language training, health promotion, and medical skills development were identified as additional strategies by which teams could support community development.* Conclusion.* Visiting volunteer groups could work in partnership with community organizations to address these barriers by providing short-term access to services, while developing local capacity in education, healthcare, and health promotion in the long-term. Future work should also carefully evaluate the impacts and contributions of such volunteer efforts.

## 1. Introduction

Decades of economic migration from Haiti to the Dominican Republic (DR) gave rise to a sizeable minority population of Haitians that represent an inexpensive source of labour for various sectors of the Dominican economy, particularly the sugar industry [1, 2]. As a result of sociopolitical discrimination, low levels of educational attainment, and denied access to identity documents, the Haitian population in the DR is significantly marginalized [3, 4]. Those employed by the sugar industry are often housed in* bateyes*, which are rural towns characterized by endemic poverty and limited access to health and social services [2].

The marginalization of* batey* populations results in poor population health status and notable community disease burdens around HIV/AIDS, tuberculosis, and malnutrition [5–7]. Specifically, a review of medical conditions treated by mobile medical clinics in the* bateyes* found that while the most common diagnoses encountered were gastroesophageal reflux disease, hypertension, upper respiratory tract infections, otitis media, and fungal skin infections, diseases unique to an indigent tropical population were also commonly encountered, including dehydration, malnutrition, parasites, and rare infections [8].

Described barriers to healthcare experienced by this group include insufficient health insurance coverage, geographic restrictions, and limited understanding of or trust in the local healthcare system arising from reports of humiliation and discrimination [2, 9]. The issues have captured the attention of volunteer groups from the United States and Canada, which visit the DR in ever-growing numbers to provide various charitable services (e.g., primary healthcare services through mobile medical clinics) [10].* Hospital El Buen Samaritano* (Good Samaritan Hospital or HBS), in La Romana, has hosted visiting groups since the early 1980s. As the third-largest city in the Dominican Republic and hub for the eastern Dominican sugarcane industry, La Romana presents one of the best opportunities to explore concerns around health and social status in batey communities in the Dominican Republic. Specifically, groups from HBS provide mobile medical clinics over one to two weeks throughout the year to a catchment population of 13,000* batey* residents across an area of 160* bateyes *(personal communication was from the Hospital El Buen Samaritano administrator Moises Sifren Juan).

Program planning and evaluation require improved evidence and data on the health status of the community, but extant literature on the subject is limited. This paper begins to address this gap by identifying key themes summarized from a community evaluation of stakeholder perceptions on the state of health determinants in the* batey* communities of La Romana. The findings of the present study represent potential areas for future exploration and service that could be targeted by visiting volunteer groups to La Romana and also will act as foundational data by which other visiting groups with efforts focused on* batey* health in the Dominican Republic might evaluate their work.

## 2. Methods

This community evaluation employed semistructured interview techniques to obtain insight from two key populations into the subject of local health determinants and the role of volunteer teams. Key informants from among local professionals that interact with visiting teams and members of the local community were interviewed. The initial step in developing an interview guide was to conduct a cursory literature search for relevant articles and health data on Haitian economic migrants residing in the DR. This consisted of a search of PubMed, a grey literature search, and obtaining health indicator statistics from Dominican Republic government databases and the Pan American Health Organisation. Authors identified valuable articles from abstracts by consensus. In partnership with local program collaborators, articles, along with relevant statistics and health data, were used to identify guiding questions which formed a framework for the development of an 18-item interview guide. Following translation, the guide was reviewed and revised by local partners for cultural appropriateness and nuance.

This guide was subsequently used in the conduct of semistructured interviews with the two key stakeholder populations described earlier: (1) members of the local population of migrant Haitian workers and (2) local healthcare providers and leaders. These groups were targeted to provide both professional and lay-person insight into various community needs and deficits relevant to volunteer activities. Data collection took place during a one-week field visit in December 2012 with community members from three* bateyes* of varied size and location and with staff at local partner institution. To ensure consistency in prompts, format, recording, and interpretation, all interviews were conducted by one of the investigators of this paper, with help from an interpreter who acted as a cultural interpreter and had received briefing on the overall project. Based on a sampling process developed by the local hospital partner, three bateyes were targeted as representative, and research proceeded as planned. The interpreter guided respondent selection using a systematic process in each of the* bateyes* (every fifth attendee at the community health clinic) and by a snowball method among hospital leadership and employees.

Interview responses were recorded electronically and on paper. Following data collection, these were collated and qualitatively analysed in aggregate by investigators for common themes, using an analysis framework based on the original guiding questions. For each guiding question, responses were coded by two different investigators for relevant themes. Any discrepancies between reviewers were resolved through consensus discussion and consultation with local partners. Following coding, key themes were identified based on frequency and targeted for inclusion in final results.

Institutional research ethics approval was obtained from the University of Toronto ethics review board (protocol ID 28419). Upon receiving approval from the University of Toronto REB, local community approval was granted by the leadership of HBS. As there was no local ethics review board in La Romana, HBS leadership granted approval to proceed with this study in the La Romana area provided that the researchers involved adhere to the terms of the REB approval granted by the University of Toronto.

## 3. Results

30 community informants were interviewed (9 male, 21 female) across three* bateyes*:* Batey* 1 (*n* = 12),* Batey* 203 (*n* = 7), and* Batey* Cacata (*n* = 11). The three* bateyes* targeted represented a collective estimated population of 3,600 inhabitants. This included* Batey* 1 which had a population of 300 inhabitants,* Batey* 203 with 300 inhabitants, and* Batey* Cacata with 3,600 inhabitants. 11 health professional informants at the local mission hospital were also interviewed and represented both administrators and direct care providers. For both groups of informants, key themes explored included community needs, health priorities, and healthcare access. Unique themes emerging among community informants centered around basic necessities, including water, electricity, food, and clothing; unique themes identified by health professional informants included specific health programs and the role of the mission hospital, as well as perceptions on disease patterns and epidemiology.

### 3.1. Community Informants

#### 3.1.1. Water

Potential sources for drinking water identified by respondents included pipe, well, and bottled water. Of these, respondents uniformly reported preferring bottled water despite its cost.

Further review of responses identifying bottled water as a preferred source highlighted several common themes as rationale: respondents believed that bottled water was more palatable and safer to drink and was also inexpensive at approximately RD $40 ($1 USD) per 19-liter bottle.

Exploring the theme of price versus quality further, responses highlighted concerns about how potable municipal water delivered by pipe was, despite the fact that it was provided free of charge. Specific to potential solutions and the role of volunteers, responses occasionally referenced a water filtration and sanitation implementation initiative jointly conducted by the local mission hospital and visiting volunteers that aimed to improve access to potable water from municipal sources in all* bateyes.*

#### 3.1.2. Electricity

While some informants reported regular and available access to electricity with reported costs in the range RD $300–$460 (approximately $7–$11 USD) per month, there were notable variations between* bateyes* that suggested infrastructure deficits. For example, respondents at* Batey* 1 reported regular access to electricity while those at* Batey* 203 reported having no access to electricity;* Batey Cacata* respondents reported partial electrification of community dwellings, with some respondents without access.

A theme arising among respondents without electric access was safety and security at night and sources of light.

Respondents identified both kerosene lamps and candles as potential lighting sources. These responses did identify slightly additional cost burden as the reported cost of kerosene and/or candles was RD $50 ($1 USD) for 3 days' use, which adds up to RD $500 monthly ($10 USD monthly).

#### 3.1.3. Food and Clothing

Respondents noted that food was not provided by the company but was purchased out of pocket. Related to this, themes identified variations in sourcing, commonly related to finances. Small, local* batey* stores (on-*batey*) and a large supermarket in central La Romana were identified as venues, and respondents stated that the* batey* store was most commonly accessed when finances were tight.

The supermarket was more highly regarded by respondents, but many of them added the caveat that accessing this venue involved greater spending to pay both for groceries and for transport (responses estimated an average of RD $2000 or US $47 in total).

When asked about clothing supply, respondents did not identify any specific concerns. Themes in responses largely discussed the duration to which clothing remained viable, which was 6 to 24 months, and plans for replacement, which involved either attending a shop in La Romana or receiving clothing via charity.

#### 3.1.4. Healthcare Access and Insurance

While some respondents did not attend Good Samaritan Hospital, others reported visiting up to 11 times in the previous year, with an average of two annual hospital visits. Uninsured individuals paid a range of fees for services of RD $1000–$5000 (approximately $23–$120 USD), depending on the nature of the visit. This included payments for primary care services, which were provided through the hospital. 19 respondents reported receiving medical insurance coverage through their company, which also covered their immediate family, with monthly costs in the range of RD $150–$400 (approximately $3.50–$10 USD) per month, depending on the plan. Insured respondents reported being satisfied with the cost of insurance. 11 uninsured respondents, by contrast, stated that hospital services were expensive.

The provision of care at the hospital was perceived as adequate by the vast majority of respondents. For responses that perceived care as being poor, expressed concerns around the quality of care included the nature of treatment being ineffective or a waste of time and money or the notion that competing needs prevented them from spending money on healthcare.

Alternative venues identified by respondents, considering time and financial constraints, included a community health promoter or going to a local Dominican* batey *clinic sometimes. These respondents perceived local* batey* clinics to be inferior to larger hospitals in La Romana but also saw them as more convenient.

#### 3.1.5. Community Needs

Exploring the relationship between education and health, specific themes around further education or training were identified, with a specific focus on what would benefit the communities. Responses identified diverse content areas, including English and French language education, health promotion and nursing skills, computer skills, and improved primary education for children, particularly protected access.

Additional themes concerned social skills, sewing, and cooking.

### 3.2. Health Professional Interviews

#### 3.2.1. Hospital Infrastructure and Programs

A common theme among responses was the important role that the mission hospital had played in providing care to the community for over twenty years. Growth was another commonly identified theme, with respondents highlighting that initial hospital construction efforts would reach completion in 2015 and that, over time, the hospital has emerged as a major medical hub for the estimated 20,000 Haitian workers living in the* bateyes*, as well as the wider La Romana community. Partnerships were another common theme identified by respondents, which noted that over 60 volunteering organizations visit annually, usually nonprofits, church groups, and academic institutions. Specific themes in the focus of these volunteer trips included both medical clinics and surgeries provided at no expense to the hospital or patients.

#### 3.2.2. Batey Health Needs and Programs

As a whole, respondents viewed medical teams favorably and uniformly identified a need for more teams in the future. They cited a number of needs that could be addressed by visiting teams on locally established community work projects. These included the construction of churches and the continued development of water filtration and sanitation systems. Specifically, the impact of mobile medical clinics was positively viewed; one respondent pointed out that this outreach effort had provided service annually to nearly 13,000* batey* residents.

Another commonly identified theme centered around the work of a local community health work (health promoter) program, which respondents identified as a potential area for collaboration with volunteer teams. Detailed descriptions provided in the responses suggested that the program recruits local* batey* community leaders into training and employment with the hospital; those recruited act as medical liaisons and first responders in their communities, providing basic medications and conducting health education. One respondent identified a deficit in the number of health promoters, however, with only 60 of 160* bateyes* served, but this deficit was explained as arising not for lack of willing promoters but was instead due to a lack of funding to employ new promoters.

The health promoter's current scope of practice is limited to taking axillary temperatures, administering first aid, checking heart rates, administering deworming medications, and health promotion.

#### 3.2.3. Healthcare Cost and Access

Themes matched those of community respondents in noting a difference in access, cost, and service variation for patients depending on insurance status, with insured patients paying approximately RD $600 (approximately $14 USD) per visit and uninsured patients paying anywhere in the range of RD $1000–$5000 (approximately $23–$120 USD) per visit, depending on the visit purpose. One respondent described other auxiliary services (such as an in-house social worker) which helped uninsured patients afford treatment. Those patients who are unable to pay are aided through establishing a payment plan or are referred to a local public hospital where the costs are generally lower.

#### 3.2.4. Disease Patterns

Respondents suggested that the most commonly reported ailments among* batey* residents were asthma, respiratory infections, and fever. There was a concern among providers that while they were largely able to effectively meet the needs of the individual patients they see in the hospital, they felt they were not meeting the health needs of the wider community.

#### 3.2.5. Perceptions of Visiting Teams

Respondents favorably viewed visiting groups and their mobile medical clinics and identified one potential focus area as being on local capacity development. Identified content themes included training for* batey* promoters, family planning education, sexual education (particularly around providing contraception and teaching its correct use), and nutritional counseling.

Additional responses suggested that groups could lend their perspective and advice on hospital processes, such as telemedicine, and develop an international physician-exchange program.

## 4. Discussion 

La Romana, like many* batey* areas of the Dominican Republic, has become the focus of an ever-increasing number of short-term volunteer efforts, particularly mobile medical clinics. To interpret our findings, we applied a social determinants of health (SDOH) framework to responses in order to identify general perturbations in key determinants that might benefit from planning efforts around visiting volunteer groups in the community. The responses provided in the previous section highlight a number of key determinants that might be addressed in order to improve individual and collective community health (e.g., limited access to healthcare, education, low income and employment, and community security). This framework guides our discussion around notable trends below. The determinants for the SDOH framework were derived from the Public Health Agency of Canada website [11].

### 4.1. Determinant: Education and Literacy

The focus of training and programs suggested by respondents varied between both the community and health professional respondents. Themes from the former group focused on skills development, such as English and French education, and access to resources, such as added nursing care. The importance of language education reflected the extant limited level of English fluency and the preponderance of Haitian Creole and, to a lesser extent, Spanish spoken in this community.

Development of community health promoter skills represented one potential avenue to increase local capacity to address a greater range of community health needs. Successes of this nature could assist new promoter recruitment efforts while allowing for evaluation of existing health promoters and the implementation of quality improvement programs.

The favorable impression of volunteer groups by respondents in La Romana suggests community willingness to share ideas and work collaboratively with visiting groups on health education and community development. Maintaining this goodwill is essential to preventing misunderstanding and maintaining a good working relationship and can be accomplished through predeparture training that focuses on cultural sensitivity, ethics, community partnership, and responsible volunteering.

### 4.2. Determinant: Physical Environments

Themes around access to water, an essential necessity, highlight several areas of concern. The perception (and likely reality) that bottled water is healthier presents a notable burden in that purchasing bottled water, no matter how inexpensively it is perceived, represents an additional financial strain on the limited finances of this population. A successful water filtration project, the inception of which was identified in certain responses, would thus both meet an essential need and mitigate the financial burden associated with bottled water purchase.

Similar themes were noted in access to electricity. In particular, lack of electricity results in inconsistent access to lighting, which has implications for community safety and evening activities. There appears to be continuing work supporting ongoing electrical infrastructure installation, especially in* Batey* Cacata, where infrastructure was being extended to new growth areas in the community.

Consensus in themes favors adequate clothing supplies with continued access to new clothing. It is worth noting that neither* batey* residents nor hospital providers interviewed mentioned donation or access to clothing as being a community need.

### 4.3. Determinant: Health Services

Despite being established as a hospital to provide for indigent residents of* bateyes*, professional respondents seemed to indicate that many of the patients treated at HBS were patients with health insurance coverage. While this might reflect an ascertainment bias, as insured* batey* citizens might be more willing to seek care, this theme parallels a similar theme noted among community respondents who reported that local hospitals were cheaper and therefore more accessible, despite quality of care concerns.

Responses on common diseases in the community were concordant between care providers and* batey* residents. Notably, few community responses identified HIV/AIDS as a common disease on the part of* batey* residents, despite high rates of prevalence documented in the literature [12]. This concurs with published factors in Rojas et al.'s study [5] which suggest that a lack of awareness or unwillingness to address HIV/AIDS is related to social stigma, lack of knowledge as to status, and limited access to testing and care providers. The stigma that limits responses has implications for both prevention and education efforts as well as screening and treatment in this population.

It is also worth noting that the DR has specific legislation that contains publically supported provisions for various allowances, such as old age, disability, survivor coverage, sickness and maternity, and family [13]. However, Haitian migrants in the Dominican Republic are not given access to these provisions under Dominican law, which is exacerbated for Haitians who lack legal documentation of status [2].

### 4.4. Determinant: Gender and Culture

Perceptions of the quality of care might well determine the willingness on the part of patients to spend time and money on seeking treatment for illness. If individuals do not believe that they will receive quality care or that the provision of the care accessible to them is not in keeping with their cultural values, they may be less inclined, able, or willing to pursue it [14, 15]. Related to the responses, this raises concerns that this disenfranchised population might choose to forgo access to care to save money, especially if they feel that the care they might receive would be inadequate. This may be influenced by cultural gender roles: do men and women perceive the necessity for healthcare similarly? And whose responsibility might it be to seek treatment for children in their care? Also, to what extent might language barriers preclude or disincentivize care-seeking behaviour?

### 4.5. Limitations and Future Work

This preliminary work aims to provide some broad representations of barriers to health among Haitian* batey* residents in La Romana. There is a possibility that the findings from the three bateyes may not be representative of the broader 160 bateyes. However, concurrence in responses with interviewed hospital and healthcare providers mitigates this somewhat.

Additional detailed interviews and analysis could be conducted with the local general hospital in La Romana and local care providers at* batey* clinics. The development of hospital and clinic indicators based on these findings could support in-depth quantitative studies. Additionally, in interviewing* batey *residents, we did not inquire into the typical salaries of* batey *workers as well as the availability and quality of food available to residents at their local stores. This information would have been useful in drawing inferences from the illnesses reported based on the nourishment available.

## 5. Conclusions

Broadly reviewing the responses against a social determinant of health framework provides a descriptive summary of the environmental, cultural, educational, and systems level barriers that may diminish the health status of* batey* populations in La Romana. Limited access to potable water, adequate food, and electricity increases community susceptibility to infectious disease and injury and exacerbates safety and security concerns. These basic issues are further exacerbated by limited access to medical insurance coverage, low family wages, and access to quality healthcare services.

In addressing these determinants, respondents have indicated that volunteer teams may have a role to play in providing services and developing capacity in these communities. This study presents an initial formative assessment of barriers to health and wellbeing in the community as well as potential strategies to address them that should be explored. Additionally, given the evolving Dominican government position on migrant Haitian communities, another worthwhile line of inquiry would also be to determine whether the Dominican government might have the resources, political will, and understanding of potential benefits for costs needed to provide programs and policies that support rather than persecute this vulnerable population. Highlighting and seizing such opportunities, while being largely theoretical at this point, would provide the most lasting improvements to the health status of the migrant worker population.

Further research will also be needed to clearly identify quantitative measures on the concerns and themes identified which can be used to direct volunteer efforts more effectively. At the heart of any redirection of effort is the community goodwill that is related to the work of volunteer teams in partnership with the local hospital, which forms the basis through which collaborative work might meaningfully address these perturbations in the root causes of ill health in these* batey* communities.
